# Hospital Annual Meetings

**Published:** 1920-03-20

**Authors:** 


### Hospital Annual Meetings.

### Elizabeth Garrett Anderson Hospital.

The Countess of Lytton presided last week over the annual Court of Governors of the Elizabeth Garrett Anderson Hospital. Owing to the purchase and presentation to the institution by Miss Aldrich-Blake and Sir Alan Garrett Anderson of two houses adjoining the hospital, one of which, however, will not be available for two years, an additional out-patient department will be provided and extra sleeping accommodation for nurses. It is proposed to arrange an eight-hour day and to separate medical and surgical cases. Only £2,219 of the £50,000 memorial fund for the endowment of beds remains to be contributed. Representatives of the various beds are to be made honorary life governors. During 1919, 1,365 in-patients were admitted to the hospital, an increase of 176 over the number for 1918, while 8,043 new out-patients, including 499 to the evening clinics, also received attention. Their attendances totalled 41,128, including 7,603 at the clinics. Total income of £15,483 was less than the expenditure by £1,059 last year.

### Prince of Wales's Hospital, Tottenham.

The Prince of Wales's General Hospital, Tottenham, the annual meeting of which was held recently, cared for 1,893 in-patients last year, as compared with 1,910 in 1918. New out-patients numbered 22,544 with 84,573 attendances, against 20,796 and 80,341 attendances a year previously. The average total cost of each in-patient per week was £2 9s. 2d., 4s. 10d. more than in 1918, while that of out-patients per attendance rose from 10½d. in that year to 1s. 1d. during the period under review. Each in-patient was resident on an average for 18½ days, the average residence in 1918 being 19 days. Subscriptions and donations last year amounted to £4,745, while £1,496 was secured from entertainments, the total income being £17,888. In addition to an ordinary grant of £3,000, the King's Fund made a further grant of a similar sum for the reduction of the institution's debt. Expenditure in 1919 totalled £17,955, and exceeded income by £67.

### Royal Waterloo Hospital.

The Lord Mayor presided over the annual Court of Governors of the Royal Waterloo Hospital, held recently at the Mansion House. In-patients to the number of 1,033 were treated last year, as compared with 833 in 1918, while 6,368 new out-patients, whose attendances totalled 21,880, were also cared for. The income for general purposes during 1919 amounted to £11,477 and the expenditure to £13,165, the failure of the income to balance the expenditure resulting in the conversion of a surplus on the revenue account to a deficit of nearly £819. The Building Committee has been reformed under the presidency of the Duchess of Albany to consider the scheme for rebuilding the nurses' home and isolation accommodation suspended during the war. Substantial progress has been made with the plans, which have already secured the preliminary approval of the King's Fund. One thousand guineas has been raised for the endowment in perpetuity at the hospital of a V.A.D. cot.

